# Correlative *In Vivo* 2 Photon and Focused Ion Beam Scanning Electron Microscopy of Cortical Neurons

**DOI:** 10.1371/journal.pone.0057405

**Published:** 2013-02-28

**Authors:** Bohumil Maco, Anthony Holtmaat, Marco Cantoni, Anna Kreshuk, Christoph N. Straehle, Fred A. Hamprecht, Graham W. Knott

**Affiliations:** 1 BioEM Facility, Centre of Electron Microscopy, EPFL, Lausanne, Switzerland; 2 CV Laboratory, EPFL, Lausanne, Switzerland; 3 University Medical Center (CMU), Department of Neuroscience, University of Geneva, Geneva, Switzerland; 4 Heidelberg Collaboratory for Image Processing (HCI), University of Heidelberg, Heidelberg, Germany; Virginia Tech Carilion Research Institute, United States of America

## Abstract

Correlating *in vivo* imaging of neurons and their synaptic connections with electron microscopy combines dynamic and ultrastructural information. Here we describe a semi-automated technique whereby volumes of brain tissue containing axons and dendrites, previously studied *in vivo*, are subsequently imaged in three dimensions with focused ion beam scanning electron microcopy. These neurites are then identified and reconstructed automatically from the image series using the latest segmentation algorithms. The fast and reliable imaging and reconstruction technique avoids any specific labeling to identify the features of interest in the electron microscope, and optimises their preservation and staining for 3D analysis.

## Introduction

For over half a century, serial section transmission electron microscopy (EM) has ruled supreme as the method for exploring the details of neuronal morphology and connectivity. Combining this method with time resolved data from the light microscope extends and enriches this approach for understanding biological mechanisms in the living organism. The available methods today, however, are time consuming, difficult, and prone to errors, motivating us to develop a new approach.

Here, we avoid the manual sectioning and imaging approach of serial section TEM and capitalize on the automated serial imaging capability of a block face method. This is used to target axons and dendrites that had been imaged previously in the live brain. We also avoid the deleterious effects on the ultrastructure from the labeling techniques that are normally employed to identify specific neurites in the electron microscope. This removes the labor-intensive process of manually segmenting features in the serial images to reconstruct in 3D the neurites of interest.

Three-dimensional (3D) imaging using electron microscopy can be achieved in a number of ways. Electron tomography (ET) uses transmission electron microscopy to image sections of up to half a micron thick, at many different angles. Nanometer resolution image series are then computed through this limited volume [1], [2]. Larger volumes can be imaged using transmission [3], [4] or scanning [5] electron microscopy of serial sections mounted on a variety of different surfaces. However, these techniques require considerable manual input for the sectioning, imaging, and also image manipulation to produce the final result.

The appearance of block face scanning microscopy has significantly increased the level of automation, reliability and speed with which aligned image series can be acquired [6]–[8]. A scanning electron beam is directed at the sample block itself, with electrons reflected from just below the surface making up the final image. After each image is taken, a thin layer of material is then removed from this face, either by an ultramicrotome incorporated into the microscope [6] (serial block face scanning electron microsocpy, SBEM), or by a focused beam of gallium ions (FIBSEM) directed perpendicular to the face [8].

Besides the advantage that these scanning microscopes can operate unsupervised for many hours to produce thousands of serial images, both techniques are capable of acquiring stacks of near isotropic voxels; the FIBSEM as low as 4 nm×4 nm×4 nm, but with a limited total volume (10,000 µm^3^), whilst the SBEM is capable of voxels with dimensions 16.5 nm×16.5 nm×25 nm across considerably larger volumes (6,000,000 µm^3^) [7].

Targeting specific cells, or regions, in tissues like the brain for EM analysis, typically requires labeling strategies to identify the structure of interest. Chemically staining endogenous markers, or genetically expressed labels using immunocytochemistry [9], as well as revealing markers injected precisely into identified cells are common [10]. These methods rely on the production of electron dense material to highlight the position of the label in the EM. Often, in the case of neuronal morphological analysis, this is done with 3,3-diaminobenzidine tetrahydrochloride (DAB). This forms a precipitate by enzymatic reactions that can cause disruption to the ultrastructure, and is further aggravated by damage to the tissue caused by the use of detergents or freezing, which are needed to permeabilize membranes and enable the reagents to penetrate more easily. Two recent exceptional studies omitted this labeling, instead using a correlative approach to find the neurons in question [4], [7]. However, these studies relied on imaging massive volumes (<1,000,000 µm^3^) of tissue, enclosing the cell body and many of its dendrites, so that the neurons could be unequivocally identified in the final image series, and matched with the previous light microscopy of the living sample.

Here, we report that instead of labeling the imaged neurites, the region containing the axons and dendrites imaged in the live brain were marked with a laser branding technique (near-infrared branding NIRB) [11], immediately after fixation, and then automatically imaged with the FIBSEM. This technique produces image stacks with near isotropic voxels that allows the use of segmentation algorithms to semi-automatically reconstruct the neurites of interest and their synaptically connected partners.

## Results

Axons and dendrites were imaged in vivo in the brains of adult mice using 2 photon microscopy. These animals expressed either GFP in excitatory cortical neurons, or tdTomato in inhibitory neurons. The imaging was carried out through a glass cranial window using previously published methods [12]. The animals were then perfused with concentrations of aldehydes (paraformaldehyde and glutaraldehyde) that optimally maintain cell ultrastructure. Importantly, such concentrations are incompatible with immunocytochemistry labeling methods due to the high degree of cross-linking, preventing adequate penetration and binding of most labels into thick slices of tissue.

**Figure 1:**
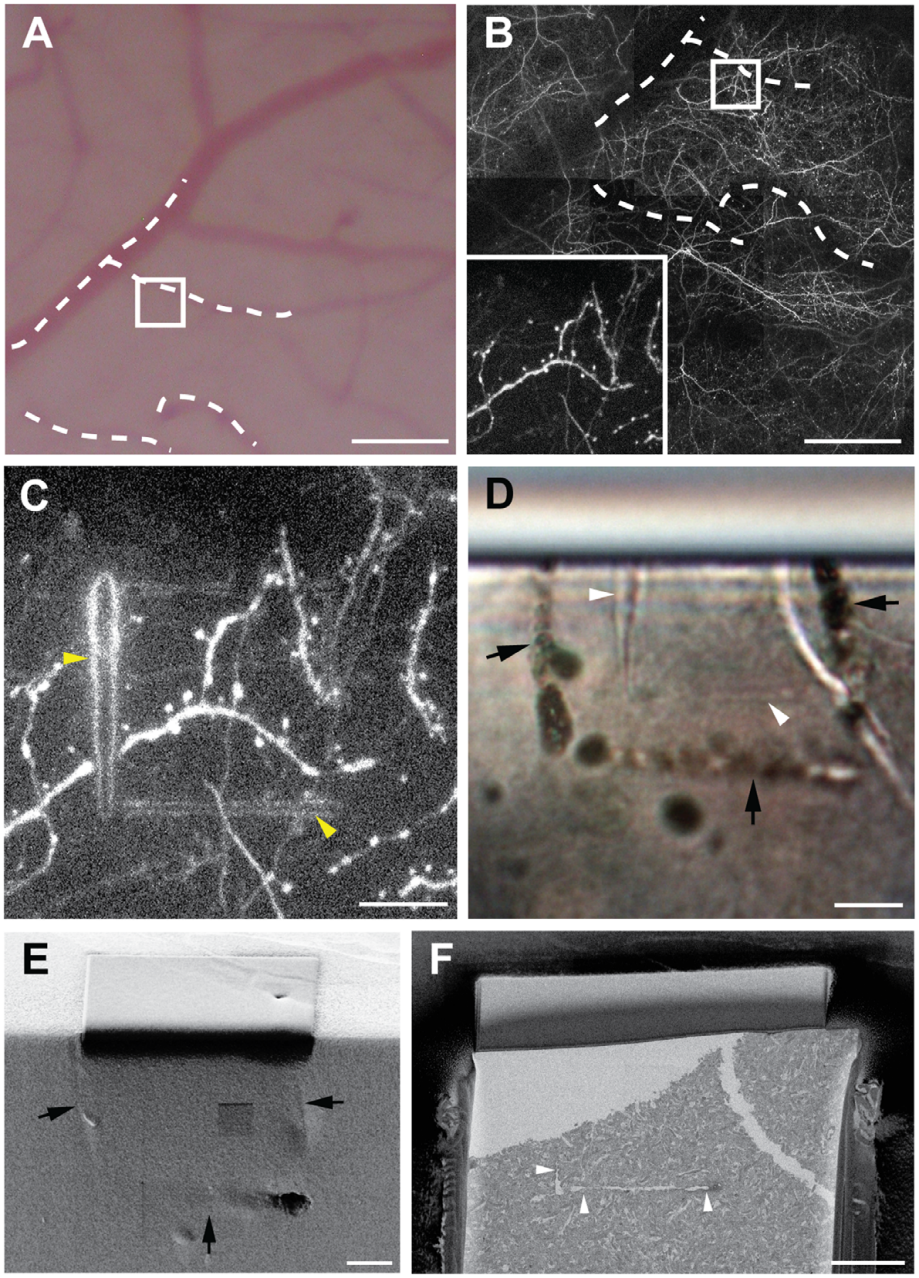
*In vivo* imaging, laser branding and tissue preparation. **A**, Cortical surface showing the vasculature on the surface of the brain. Dotted lines indicate the blood vessels that can also be seen as dark shadows in the 2PLSM (**B**), with the white square indicating the region imaged at higher magnification (inset). After fixation and sectioning this region (**C**) was then laser branded, and reimaged using 2PLSM. These branding marks were visible (**D**) in the resin block (indicated with white arrow heads) without any further enhancement. Their position is also highlighted with laser etching on the surface (black arrows) that can be seen in the FIBSEM (**E**). This indicates the region to be imaged (**F**) so that imaging and milling will capture the branded region (white arrow heads). Scale bar in **A** and **B** is 100 µm, and 10 µm in (**C**–**F**).

After perfusion fixation, the imaged brain region was then sliced tangentially to the cortical surface, parallel to the imaging window and focal plane of the 2-photon laser-scanning microscopy (2PLSM). This ensured that the first few sections contained the region of interest, as well as the brain surface vasculature, as it was seen through the cranial window (**Fig. 1A**) in the live animal. This vasculature provided essential landmarks with which the fluorescent neurites of interest could be located in the fixed sections (**Fig. 1A, B**). Capturing the vascular pattern is critically important to pinpoint imaged axons and dendrites, particularly in neural tissue that shows high densities of fluorescent neurons.

Once the exact segments of neurites were located, and orientated to the same position as seen in the live animal, the 2PLSM was used to brand fiducial marks around this region of interest (**Fig. 1C**). We used line scans with sufficient laser power (see Methods) to burn stripes at the same focal plane as the neurites. The stripes were visible in the 2PLSM through an increase of the autofluorescence at the edges of the line scans (**Fig. 1C**). Previously these marks were then labeled for EM using a photoconversion step with DAB. However, this was unnecessary, as the marks themselves were clearly visible in the resin-embedded section (**Fig. 1D**). The slice of tissue was, therefore, immediately treated with a standard EM protocol for tissues and cells, using heavy metal stains that gave a moderate contrast to all membranes. However, the labeling was not too intense, leaving 60 micron thick resin embedded sections sufficiently transparent for the branding marks to still be seen with a transmitted light microscope (**Fig. 1D**). In this embedded material these marks were the only indication of the neurites’ position, since at this stage any fluorescence had been completely lost.

Vizualising the laser marks meant that the resin embedded section could be trimmed in the ultramicrotome with glass knives, and extraneous material removed, leaving the region of interest approximately 5 µm below the surface. Using a laser dissection microscope, the position of the laser branding marks was etched onto the surface. This left an indentation that is visible with the stereomicroscope fitted to the ultramicrotome, and also in the electron microscope (**Fig. 1D, E**). These landmarks eliminate the need for repeatedly removing the block from the trimming device to check the position of the region of interest inside the tissue. Their visibility in the FIBSEM (**Fig. 1E**) was also used to align the block for final milling and imaging.

The ion beam milled parallel to the block face, approximately in the same plane as the focal plane of the 2PLSM, and also parallel to the plane of the brand marks. As these were about 5 µm below the surface only a limited amount of material was imaged before the neurites of interest began to appear. The microscope recorded images with a horizontal field width of approximately 12 µm, and height of 10 µm with a pixel size of 6.0 nm for GFP labeled excitatory neurons and with a field view approximately 15 µm×15 µm with a pixel size of 6.0 nm for tdTomato labeled inhibitory neurons, respectively. The FIB beam was set to mill approximately 6 nm from the surface of the block after each image was taken. The z depth for each image was calculated more precisely, post hoc, by using the cylindrical diameters method [13], giving a value of 7.78±0.86 nm for GFP labeled tissue and 8.71±0.74 nm for tdTomato.

**Figure 2:**
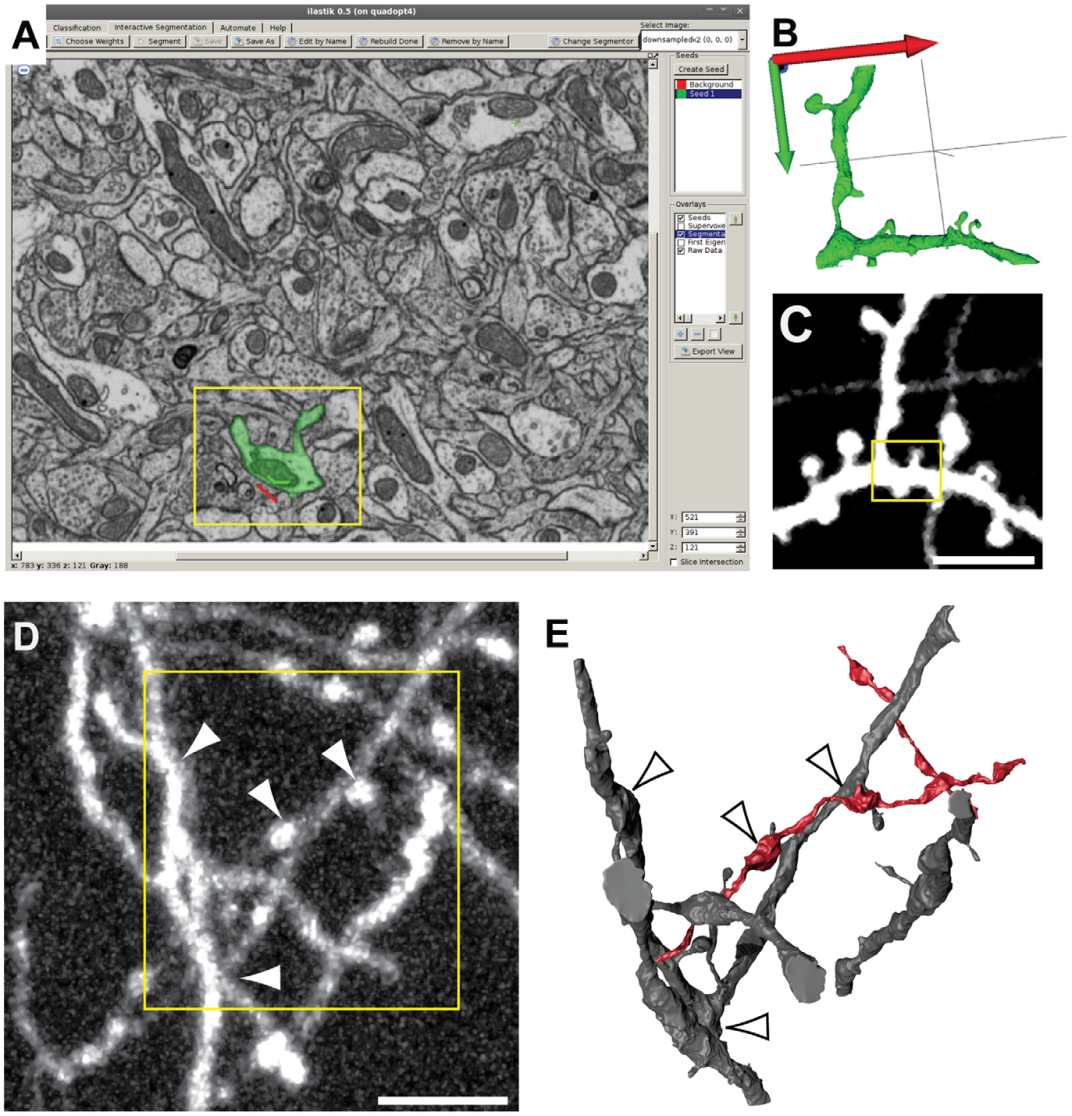
Interactive segmentation of neurites. **A**, The ilastik software allows users to select regions for segmentation (yellow box), and compare the generated 3D model (**B**) with the *in vivo* image (**C**) of the neurite of interest. A GFP dendrite from a layer 5 pyramidal neuron is shown in **C**. The method can also be used with other fluorescent markers, such as tdTomato (**D**), here expressed in GABAergic axons and dendrites in a different animal. When the density of neurites (**E**) is high the neurites can still be distinguished. Panel **E**, shows the reconstructed axons (red) and dendrites (grey) that are shown in the in vivo image in **D**. Scale bar in **C** and **D** is 5 µm.

**Figure 3:**
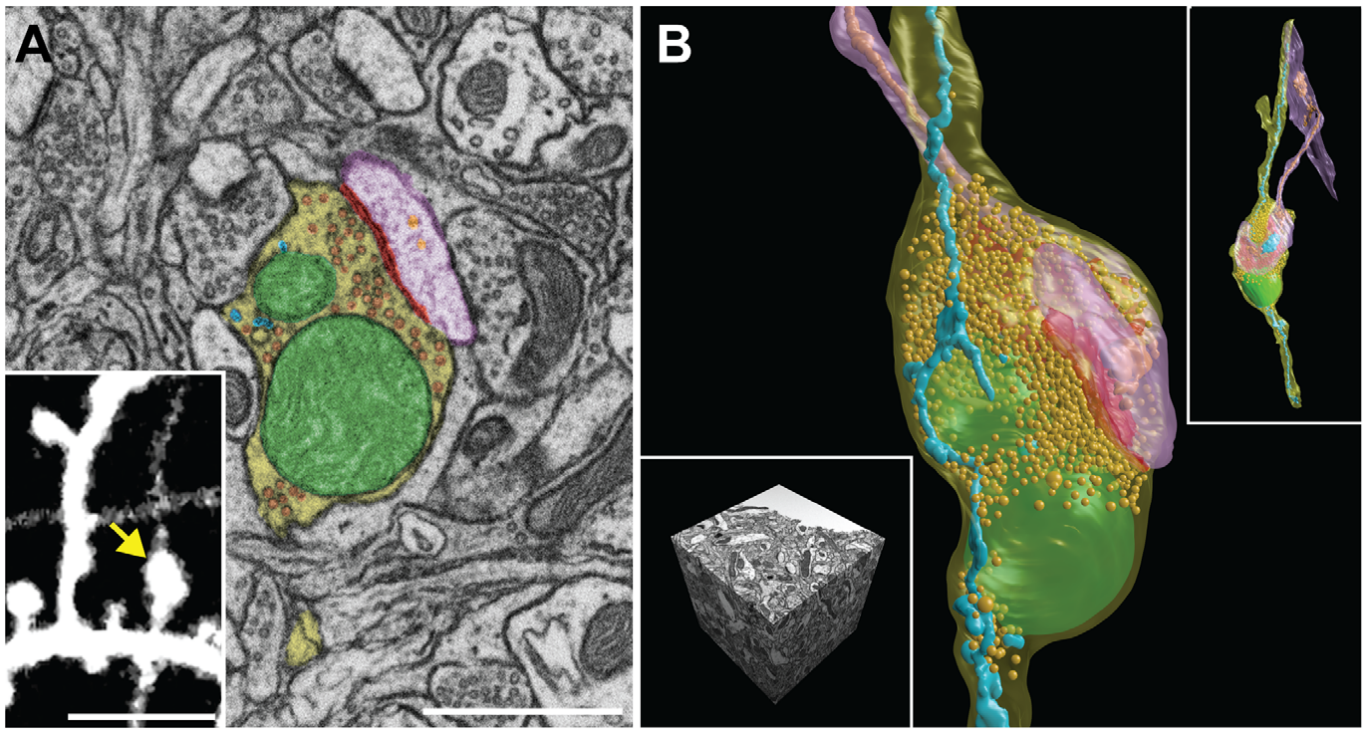
Manual reconstruction of a bouton its synaptic partner, and all membrane organelles contained. **A**, **B**, The FIBSEM image series can be used to segment *in vivo* imaged structures (**A**, inset) including all their organelles: axonal bouton – yellow, mitochondria – green, synapse – red, synaptic vesicles – gold, endoplasmic reticulum – blue, dendritic spine – pink, dendritic endoplasmic reticulum – orange. The reconstruction is made from an image volume (6.4 µm×8.0 µm×5.0 µm) (**B**, inset left) that also includes the synaptically coupled dendritic spine (**B**, inset right). Scale bar in **A** is 1 µm, and in **A** (inset) is 5 µm.

This small z depth between images ensures small changes in structural information between each one, giving the best opportunity for 3D segmentation algorithms to reconstruct the neurites. We used the interactive segmentation tool [14] inside the ilastik software (www.ilastik.org) to reconstruct neurites from the series. Given user input, this tool performs a weighted watershed segmentation on a superpixel graph of the 3D stack. Specifically, the user needs to mark the inside (object) and the outside (background) parts of the area of interest (**Fig. 2A**). Provided that its boundaries, ie. the membranes, are well stained, only a few (2–8) seeds are required to fully reconstruct an object such as the neurite shown here (**Fig. 2**). This number can be lower if the user is only interested in the overall topology and direction of the neurite, rather than capturing all protrusions, like spines from the dendritic shaft. The one-time construction of a superpixel graph renders the segmentation process fully interactive and allows the user to immediately assess the results in 2D (**Fig. 2A**) and 3D (**Fig. 2B**). These properties make ilastik especially suitable for quickly locating the labeled neurites in the EM image stack: only a few clicks are needed to establish if a given object continues in the same direction or branches in the same way as the labeled neurite in the *in vivo* 2 photon image (GFP labeled neurites, **Fig. 2C**; and tdTomato labeled neurites, **Fig. 2D**). After the labeled neurite candidates are found, their segmentation can be refined by additional object and background seeds, correcting the areas where segments bled into neighboring objects or were cut short by a heavily stained internal membrane. The total time for the basic reconstruction of the dendrites and axons shown in **Fig. 2E** was approximately 2 hours (stack containing of 1505 images). This is a fraction of the time that would be required if this task was to be undertaken by manually segmenting each feature in the serial images.

A detailed manual segmentation was carried out on a single glutamatergic (presumed) bouton seen in the live brain (**Fig. 3** **)**. Using immunocytochemistry methods to identify such a structure would leave its morphology compromised, with poorly preserved membranes and features concealed by the labeling. Here, with no specific labeling, the quality of the near isotropic imaging allows all the membranes and organelles to be seen. The size of the total imaged volume at this resolution also contains the synaptically connected dendrite that has been included in the model (**Fig. 3**). The entire reconstruction was made from a sub-volume of the original image stack with dimensions of 6.40 µm×8.0 µm ×5.0 µm. A single section of this thickness would be impossible to image with any transmission electron tomography (ET) method. Targeting a specific site like this for ET would also be hindered by the use of thin sections manually cut and placed onto supporting grids where the metal bars may obscure the required regions. Achieving such a 3D resolution would also not be possible with serial section TEM in which section thicknesses are usually around 50 nm.

## Discussion

The correlative light and FIBSEM method shown here completely avoids the typical problems encountered with the approach of serial sectioning TEM. Eliminating any labeling protocols used to help identify the neurite of interest in the EM means that markers giving only a weak reaction with immunocytochemistry can still be used. No steps are needed to enhance the label seen with light microscopy so that the ultrastructure is maximally preserved. Section folds and dust contamination, which obscure imaged regions in TEM, are avoided; and broken grids or missed sections are no longer an issue. This new approach shows a high degree of automation, as well as a quality and continuity of image series that allow 3D segmentation algorithms to be used. This rapid rendering of the objects in 3D with these semi-automated approaches becomes a prerequisite for identifying with certainty the elements that were previously imaged with light microscopy. Without any identifying label within the imaged neurites, these reconstructions are used to confirm the correct ones have been chosen. The task would not be possible with manual segmentation in such a short period. Taken together, the entire method makes up a new workflow, significantly reducing the time for the imaging, identification, and reconstruction of previously imaged cell structures.

## Methods

### 2 Photon Laser Scanning Microscopy (2PLSM)

All animal work was performed according to the Swiss Federal Laws on Animal Experimentation administrated by the Veterinaire Cantonale Geneve (AH). The procedures for in vivo imaging of fluorescent neuronal structures in the cortex of transgenic mice have been described previously [12]. Briefly, a small craniotomy was produced spanning approxiamately 3 mm in diameter above the somatosensory cortex. The island was replaced with a cover glass (#1 thickness), generating a closed optical window. The window was sealed using dental cement covering a small edge of the glass and the exposed skull. After approximately 7 days imaging was started. Transgenic mice expressed GFP in cortical pyramidal cells (line M [15]), or tdTomato in cortical inhibitory interneurons. The latter was obtained by crossbreeding of the B6.Cg-Gt(ROSA)26Sor^tm14(CAG−tdTomato)Hze^/J-line (Alan Institute for Brain Science; obtained from Jackson Laboratories [16], with the B6.129P2-Pvalb^tm1(cre)Arbr^/J-line (gift from C. Petersen, EPFL; generated by S. Arber [17]). During imaging the animals were anesthetized with an intraperitoneal injection of ketamine (0.13 mg g^−1^ body weight) and xylazine (0.01 mg g^−1^).

A custom-built 2PLSM, equipped with a femtosecond-pulsed titanium-sapphire laser (Chameleon Ultra II, Coherent) and a 40× water immersion objective (0.8 NA, Zeiss) was used for imaging. Both low-resolution image stacks (512×512 pixels; 3.2 µs pixel dwell time; 0.3 µm/pixel; 3 µm steps) of the region of interest and high-resolution image stacks (512×512 pixels; 3.2 µs pixel dwell time; 0.09 and 0.05 µm/pixel; 1 µm steps) of selected axons and dendrites were collected and correlated to the unique vascular pattern in the surrounding region (**Fig. 1** **A, B**). GFP-expressing neurons were imaged at λ = 910 nm, tdTomato-expressing neurons were imaged at λ = 1040 nm. Bright field images of the vasculature were taken through the oculars of the microscope immediately after imaging and just prior to fixation (Nikon Coolpix 4500).

### Tissue Preparation

The anesthetized animal was fixed by cardiac perfusion of 10 ml of isotonic PBS immediately followed with 200 ml mixture of 2.5% glutaraldehyde and 2% paraformaldehyde in 100 mM phosphate buffer, pH 7.4 (12 ml min^−1^) using a peristaltic pump. Two hours after perfusion the brain was removed and 60 µm thick vibratome (Leica VT1200S; Leica Microsystems, Vienna, Austria) sections were cut through the somatosensory cortex. These were imaged at low magnification (×20) in wide field, using phase contrast to highlight the vasculature. The images were superimposed and aligned according the vascular pattern [18]. The vascular pattern was then used to identify the section containing the region that was previously imaged in vivo. This section was temporarily mounted in PBS, for laser branding, inside an imaging chamber built using a Parafilm spacer under a coverslip.

### Laser Marking Regions of Interest

Laser fiducial marking of the selected dendrite of interest were carried out in the 2PLSM as described by Bishop et al [11]. The laser was tuned to λ = 810 nm and its power was modulated to reach ∼300 mW at the back focal plane of the objective. Typically we used 512 to 2,500 line scans (2 ms/line) under visual control, until autofluorescence started to appear. To distinguish the correct orientation of the neurites of interest within the brain section, typically two asymmetric shape laser marks were burned: large trapezoid shape of size approximately 50–100 µm×50–100 µm containing the second smaller rectangle (25 µm×25 µm) right around the selected dendrite of interest. To visualize the exact position of the marks, image stacks of 1 µm steps were collected at λ = 910 nm, using regular frame scans (512×512 pixels; 3.2 µs pixel dwell time; 0.3 µm/pixel or 0.09 µm/pixel) before and after the laser marking (**Fig. 1C**).

### Embedding Section for Electron Microscopy

After laser marking, the coverslip of the chamber was removed, the section washed in cacodylate buffer (0.1 M, pH 7.4), and postfixed for 40 min with 1.5% potassium ferrocyanide, and 1% osmium tetroxide, followed by 40 min in 1% osmium tetroxide alone, each in 0.1 M cacodylate buffer, and then 40 min in 1% aqueous uranyl acetate. Then, sections were dehydrated in graded alcohol series, and finally infiltrated with Durcupan resin overnight (Fluka, Buchs, Switzerland)). The sections were flat embedded between glass slides in fresh resin and polymerized for 24 h at 65°C. This leaves the laser marks visible in the resin sections without the need to enhance them with a photo-conversion step.

### Block Preparation for the FIBSEM

The resin embedded section was separated from the glass slides and using a light microscope the laser marks were identified. Their position was marked using a laser dissecting microscope (Leica LMD) to etch the shape of the NIRB marks on the surface of the resin (**Fig. 1D**). This ensured that the position of the imaged neurites could easily be seen during the subsequent trimming steps in the ultramicrotome.

This entire region was then glued to a flat, 1 mm thick, slab of blank resin. This gave a solid support for the region of interest that could be viewed easily with the transmitted light microscope, and also easily held in the jaws of the ultramicrotome clamp for trimming.

Using a transmitted light microscope, with a calibrated focussing stage indicating the height in microns, the NIRB marks were imaged and their depth from the surface measured. This block was then trimmed using glass knives until around 5 microns remained between the surface and the NIRB marks. During this process the block was repeatedly removed from the ultramicrotome and the surface to laser mark distance measured, taking care to only remove small amounts (around 5 microns) each time.

At the final stage of trimming the laser dissection microscope was used to replicate NIRB marks onto the trimmed block surface (**Fig. 1D**). This final block was then photographed using a transmitted light microscope to show the position of the NIRB marks inside the resin embedded tissue, as well as the etched laser marks on the surface (Fig. 1D). This was then mounted onto an inclined (45°) SEM aluminum stub using conductive carbon paint, and coated with a 30 nm layer of gold (Cressington vacuum evaporation system, USA). This type of stub ensured that the block could easily be orientated in the FIBSEM so that the ion beam milled the block face parallel to the NIRB marks.

### Milling and Imaging

The block was imaged inside a Zeiss NVision 40 FIBSEM microscope (Carl Zeiss SMT, Germany), which combines high-resolution field emission SEM with a focused beam of gallium ions. Ion beam was used in conjunction with a gas injection system to deposit a thick (∼1 µm) layer of platinum on the top surface of the sample above the region of interest to reduce the FIB milling artifacts.

At low magnification, and using secondary electrons for imaging (5 kV, 0.5 nA), the block was oriented so that the chosen region of interest and the side of the block to be imaged is facing the operator and that the face to be imaged lies parallel to the milling beam (electron beam is oriented at 54° to this face). The etched laser marks on the top surface of the block help to locate the region of interest (**Fig. 1E**).

To prepare the initial imaging face (100 µm×100 µm in size), an ion beam current of 13–27 nA at 30 kV was applied. This removed a narrow band of resin from the face of the block, which was then imaged in the backscattered imaging mode to see the tissue inside (**Fig. 1F**). Next, a protective layer (∼ 1 µm) of carbon was deposited onto the surface of the block, above the region of interest. A current of 700 pA was then used to finely mill this region of the block, within which the final images were taken. To prevent the re-deposition of the material after ion milling, the milled area was always much larger (50 µm×50 µm) than the imaged area in both the x and y directions. The area was milled until no milling artifacts (white streaks or ‘curtains’ appearing vertically in the image) can be seen on the face. To reduce the risk of the block face drift during imaging and subsequent image misalignment, the microscope was left for at least 2 hours to equilibrate any thermal changes.

For the final serial imaging, an acceleration voltage of 2 kV, with a current of between 340–400 pA and dwell time of 10 µs/pixel were used so that total time per milling/imaging cycles was maintained below 2 minutes. For the GFP labeled excitatory neuron dataset, the images were collected at a magnification of 6.08 nm/pixel, and with the total image size of 2048×1536 pixels (∼3 MB/image), which corresponds to a field size of 12.45 µm×9.34 µm. The milling depth after each image was 7.78±0.86 nm as determined by the cylindrical diameters method [13]. For the tdTomato labeled inhibitory neuron dataset, the images were collected at a magnification of 6.00 nm/pixel, and with the total image size of 2499×2506 pixels (∼6 MB/image), which corresponds to a field size of 14.99 µm×15.04 µm. The milling depth between each image was 8.71±0.74 nm as determined by the cylindrical diameters method [13]. Briefly, average section thickness was estimated by averaging the ratios of the diameter of mitochondria sectioned longitudinally within imaging plane to the number of sections they span (36 independent measurements in three different z-depths across the image stack).

### Image Processing and 3D Reconstruction

The individual images acquired by FIBSEM microscope were combined into a single image stack file: made of 1275 images for GFP and of 1505 images for tdTomato labeled neurons,. Both datasets were aligned in the Fiji software package (http://fiji.sc/wiki/index.php/Fiji) using the StackReg registration plugin with transformation parameter set to ‘translation’. The total volume used for the 3D reconstruction was 12.449 µm×9.337×9.918 µm for GFP labeled and 14.994 µm×15.036 µm×13.109 µm for tdTomato labeled datasets. Segmentation of the structures of interest, and 3D reconstruction was achieved using the Ilastik software (version 0.5.06; http://www.ilastik.org) and its seeded watershed segmentation function. After opening the image stack in the ilastik program, the set of features to identify the boundaries were computed by selecting a ‘classification’ tab and then the ‘select feature’ button. In the feature selection dialog, we then computed “texture” features, as they contained the Eigenvalues of the Hessian matrix that served as a good membrane detector. The scale of the features was set to sigma 1.0 (“medium” in ilastik). After feature computation, we switched to the “Seeded watershed” tab and selected the previously computed “Eigenvalue of the Hessian matrix of Gaussian 1 Channel 2” as input weight. The “input weight” selection dialog also required specifying whether boundaries in the weights are indicated by “darkness” or “brightness” which we set to darkness. Next we set the background bias parameter to 0.95 and lines, or seeds, were drawn to mark the inside of required features, in this case axons and dendrites. The background or outside of the feature of interest was also marked with another hand drawn line. Activating the ‘segment’ button started the segmentation process through the entire stack, and provided different views to assess the result. Iterative improvement was possible by adding further foreground or background seeds in regions where the segmentation was not perfect. The final 3D model was displayed by rendering all contours of the segmented structure (right-click on the ‘segmentation’ overlay). This model was exported as a 3D object (wavefront.OBJ file) for postprocessing. To reduce memory consumption, the images were downsampled by a factor of 2. The final visualization of the reconstructed 3D model was rendered in the Blender software (version 2.57; Blender Foundation; http://www.blender.org).
